# American Orthopedic Association

**Published:** 1891-11

**Authors:** 


### AMERICAN ORTHOPEDIC ASSOCIATION.

FIFTH ANNUAL SESSION, HELD AT WASHINGTON, D. C., SEPTEMBER 22, 23, 24, AND 25, 1891.

(Abstract of the proceedings.)

### ORTHOPEDIC SURGERY AS A SPECIALTY.

Dr. A. B. Judson, of New York, in the president's address, said that orthopedic surgery is specially the domain of physical demonstration where subjective symptoms give place to objective signs, where treatment is chiefly mechanical, and where results are recorded in degrees of a circle and fractions of an inch. It exists and thrives as a specialty, because the general practitioner concurs with the public in committing patients who, from the nature of the case, generally recover with some deformity and disability to the care of experts.

CRURAL ASYMMETRY AND LATERAL CURVATURE.

Dr. H. L. Taylor, of New York, described two instances in which the leg was two inches and one and one-eighth inches short respectively. Both cases were in young women. The short limb was larger and stronger ; the shortening was chiefly below the knee, and there was no lateral curvature.

Dr. A. Hoffa, of Wurzburg, Germany, described a specimen which proved that in one instance the shortness was due to union of the neck and shaft of the femur at an acute instead of an oblique angle.

Dr. F. Beely, of Berlin, illustrated with specimens of lateral curvature and ingenious models the changes which occur in the bodies of the vertebra preceding rotation, explaining how the para-spinous sulcus is shallow and broad on the concave, and deep and narrow on the convex sides, a condition which is reversed in the lumbar region by the absence of ribs.

TREATMENT OF HIP DISEASE.

Dr. Phelps said that traction and fixation should be enforced to prevent destruction by intra-articular pressure. Ankylosis is the result, not of fixation, but of disease. The patient should be put to bed from three weeks to four months, and should then wear a lateral traction fixation splint, which was exhibited. Children under three years are placed in a plaster of Paris portable bed, which was also shown.

Dr. Wirt exhibited a new device for traction, in which the force of the lever is changed into rectilinear instead of circular motion without key, screw-driver, wrench, buckle, or strap.

Dr. R. H. Sayre, of New York, said the invention gave accurate and easy adjustment in the direction of traction, but in the direction of relaxation the control was defective.

Dr. Taylor practiced rest in bed with traction in the acute stage, to be followed by a splint which allows locomotion.

Dr. Sayre thought but few cases required lateral traction. When the inflammation had ceased, he applied passive motion. If the pain and tenderness following last more than twenty-four hours, the passive motion had not been rightly used.

Dr. E. M. Moore, of Rochester, believed that a joint only moderately inflamed demands motion. He employed traction with a certain amount of motion.

### CONGENITAL DISLOCATION OF THE HIP.

Dr. PHELPS exhibited apparatus for the treatment of this affection, and described his method and its results.

Dr. E. H. BRADFORD, of Boston, had modified the apparatus in previous use by adding an appliance with which the patient is allowed to walk about. The joint is thus protected as in convalescence from hip disease. These appliances he had made of aluminum for the sake of lightness.

Dr. C. C. FOSTER, of Cambridge, said the best recorded result had been obtained by Dr. Buckminster Brown, whose patient was treated by mechanical means in bed.

Dr. A. HOFFA had operated by deepening the acetabulum, which is practicable from the thickness of the pelvis at this point. At first he sewed a periosteal flap over the trochanter, but this is unnecessary. Two months ago he examined his first case, two years after the operation, and found a movable joint, freedom from the characteristic gait, and absence of lardosis.

Mr. HOWARD MARSH, of London, divided these cases into (1) those in which the bone slips about on the wall of the pelvis, and (2) those in which it is fixed. The majority belong to the second class, and in these operation is useless, but is more properly applicable to those cases of the first class in which the head is high up and movable. The anterior position is the most favorable, because lordosis, which depends on the backward displacement of the head of the femur, is absent.

### MALIGNANT DISEASE AND POTTS' DISEASE.

Dr. JUDSON reported three cases in which Potts' disease and malignant disease of the vertebra had been confounded by himself and other observers. In one the diagnosis was made *ante mortem.* The patients were four and a half, thirty-five, and forty-two years respectively. The chief diagnostic points are: (1) Deformity present in Potts' disease, absent in malignant disease ; (2) local disability ; and (3) local pain ; both absent in Potts', and present in malignant disease.

Dr. WILLARD had seen two cases in which his diagnosis was confirmed *post mortem.*

Dr. GIBNEY reported a case in a man of forty years, in which he and others had been baffled in diagnosis. There was sarcoma of the fifth and sixth cervical vertebræ.

Mr. MARSH related the case of a child which was extremely difficult to diagnosticate, and which proved to be malignant in character.

SYPHILITIC POTTS' DISEASE.

Dr. RIDLON said that in this form the onset is more rapid, the pain and disability greater, the kyphosis sharper in outline, and abscesses often appear before deformity. If recognized lesions of hereditary or tertiary taint are present, treatment should be by large doses of mercury and iodide of potassium.

POTTS' DISEASE IN THE OLD.

Mr. MARSH had observed instances of suppurative tuberculosis in the metacarpus, tarsus, testis, cervical glands, knee and hip, in eight patients between sixty-three and seventy-three years. But senile tuberculosis of the spine is most rare. He had seen two cases. The patients were sixty-four and sixty-five years respectively. The College of Surgeons of London possessed an osseous specimen of the action of senile tuberculosis of the upper cervical vertebra. In his "Studies of Old Case Books," Sir James Paget had recorded a case of Potts' disease in a gentleman of fifty-five attended with angular curvature.

Dr. SAYRE recalled the case of a patient, *æt.* fifty-five, who recovered from Potts' disease with paraplegia and abscesses.

POTTS' DISEASE AND PREGNANCY.

Dr. T. H. MYERS, of New York, had collected twenty-five cases of labor in fifteen patients recovered from Potts' disease. In no instance did caries recur. But of seven cases in which the disease developed during pregnancy, three died, and three were left paraplegic. Normal parturition often follows in cases of deformed pelves, whose measurement would indicate that it was impossible. These patients should be examined by the obstetrician early in gestation.

Dr. TAYLOR knew of many cured patients whose marriage had been followed by the birth of healthy children.

Dr. G. W. RYAN, of Cincinnati, thought it was a question of allowing the tuberculous to marry. He knew of married women, deformed by Potts' disease, who had borne and raised healthy children.

Dr. LEE said that one of his patients with a large lumbar kyphosis had borne twelve children who, with the mother, are all in good health. He thought Potts' disease, even in the lumbar region, rarely produced narrowing of the pelvis.

### PARAPLEGIA IN POTTS' DISEASE.

Dr. Brackett said that relief from paraplegia may be confidently expected from continuous extension and fixation even in cases of eighteen months' standing. This should be continued for some time after recovery.

Dr. Young reported two cases of complete recovery in which there had been absence of sensation, a feature aways of grave import.

Dr. Shaffer referred to a case in which the autopsy showed that a portion of the eighth dorsal vertebra had nearly cut through the cord, leaving but a slender thread.

Dr. Hoffa said that in these cases the spine should be put absolutely at rest. He had collected thirteen operations within the vertebral canal. Two died at once, two recovered, and would, perhaps, have done so anyway. In the others there were immediate good results, but relapses soon occurred. The operation has no great future before it, and should be limited to those cases in which the processes alone are affected.

Mr. Marsh said that paralysis rarely depends on the pressure of an abscess, but (1) on softening of the core, (2) pressure of a displaced sequestrum, and (3) most common on pressure from exudation. He would only operate after thorough trial of rest.

Dr. Willard said that we could not absolutely diagnosticate the cause. When there are extensive inflammatory deposits about the arches, laminectomy may relieve the posterior pressure and allow expansion of the cord.

Dr. Lee said that in all cases of this form of paraplegia suspension would materially hasten recovery.

### ABSCESSES IN POTTS' DISEASE.

Dr. Townsend thought that, as a rule, these abscesses should not be opened. In some cases aspiration should be done, and in others the cavity should be opened and drained to prevent sepsis and danger to life. His views were based on the history of 380 patients, seventy-five of whom had abscesses.

Dr. Vance advocated aspiration, repeated as often as fluid is detected. In this way he cures three out of five cases. The depot is thus kept small, and the extent of subsequent operations, if necessary, is limited.

Mr. Marsh had rarely obtained a good result by the use of the aspirator. He said that in his observations it is best to open freely, evacuate thoroughly, and then apply pressure to assist in closing the cavity.

Dr. Willard would let dormant and caseating foci alone; liquefacting collections he would aspirate and inject with iodoform emulsion, and if true pus were present he would incise, wash out with sublimate solution, and avoid undue manipulation, which might cause fissures which would let the tuberculous poison into the system. He would then suture the incision and inject iodoform and boiled olive oil.

Dr. Shaffer would not say that incision was never advisable, but generally it is wrong to open one of these abscesses. A very large abscess cannot be washed out, and its disappearance may be confidentially expected, especially if efficient mechanical treatment is practicable.

Dr. Myers said that it was proven (1) that it is impossible to completely remove bacilli from the abscess cavity, and (2) that bacilli-infected wounds at times heal primarily. Infection is more imminent after incision, because the wound lays open channels of absorption.

### WIRING THE VERTEBRAL PROCESSES.

Dr. Hadra suggested that the spinous processes at the seat of the disease be exposed, and then firmly wired together, to secure rest and prevent deformity. The operation, as he had performed it for fracture of the cervical spine, was extremely simple and effective.

Dr. Sayre thought the wires would not bear enough force to remove the weight from the vertebral bodies, and that outside protection would be necessary to prevent lateral and rotary disturbance.

Dr. Judson thought it was a question whether wiring was applicable through the long periods in which consolidation is delayed. Intolerance of the skin always prevents such pressure as we would like to make on the kyphos. The method proposed circumvents this difficulty.

Dr. Moore said it was a most simple and harmless procedure, and, notwithstanding the theoretical objections, he would accept the first favorable occasion to try it.

PROGNOSIS AND TREATMENT OF POTTS' DISEASE.

Dr. Ketch had learned from seventy-five cured cases that in length of treatment and degree of deformity the upper region of the spine is most favorable, and the middle least of all; that paraplegia more frequently accompanies disease in the upper than in the lower regions, and that cases of traumatic origin recover sooner than those of tubercular origin. Sudden deaths sometimes occur in cervical caries from interference with respiration.

Dr. B. Bartow, of Buffalo, said that the earliest important sign in the dorsal and lumbar regions is lateral curvature dependent on nervous tenderness. Apparatus should be constructed to oppose the rotation accompanying the lateral curvature, as well as the antero-posterior deformity. He used the plaster-of-Paris jacket applied to effect the above objects.

Dr. Foster said that extension in *bed* is the best method in the acute stage. Extension should be made by light weights, the cords leading over the head and foot of the bed, and attached to waist-belts, chest-belts, and head-straps.

Dr. Ryan said recumbency was the ideal treatment, but it is in many cases impracticable. He had found splint plaster jackets efficient after the acute stage.

Dr. Lee said that many years ago, when the plan had fallen into entire disuse, he was the first to adopt suspension from the practice of Dr. J. K. Mitchell. The apparatus was Le Vacher's head support and jury-mast, attached to a chair or go-cart, or to a door-way swing.

Dr. Sayre said that in the cervical and upper dorsal region a metal posterior splint, supported on the pelvis, should be used with a jury-mast, and in the lower dorsal and lumbar regions, a plaster-of-Paris jacket with a jury-mast. Recumbency should be practiced in the acute stage; children should not be placed in the wire cuirass.

Dr. Ketch had been disappointed with the plaster of Paris and jury-mast in the cervical and upper dorsal regions. He recommended the Taylor apparatus and chin-piece. In the lumbar region almost any supporting apparatus will secure a good result.

Dr. Taylor said that the antero-posterior lever secures rest and protection, and combats deformity. Old and neglected cases are especially amenable to treatment, just as ankylosis is later and rarer than is generally supposed. Abscesses and paraplegia do not forbid a favorable prognosis.

Dr. Bradford said that the plaster-of-Paris jacket was the readiest method, but had its disadvantages; that a steel brace gave better support, but demanded more skill and care; and that recumbency was the surest way to prevent deformity, but, as a rule, was impracticable for the long periods covered by the disease.

### RHEUMATIC SPONDYLITIS.

Dr. Ryan said that this rare affection should not be confounded with rheumatoid arthritis of the spine. It is usually accompanied by rheumatic manifestations elsewhere. In the early stage the symptoms resemble those of tubercular spondylitis. Later the deformity is not angular, but resembles that of senile kyphosis. Treatment should be directed to the relief of pain by support, cautery, and medication. In the chronic form, when pain has lessened, mobility should be encouraged by passive motion.

Dr. Hoadley deplored the confusion which is found in the nomenclature of these conditions, which produce such a variety of results. He thought both rheumatism and osteo-arthritis were microbic diseases. If ligamentous structures interfere with motion, passive motion was proper.

Dr. Lee was reminded of a case which was at first thought to be spinal myalgia, but which proved to be gouty disease of the cartilages, and infrequent affection. Apparatus afforded relief, but, of course, not cure.

Dr. Ryan said that gouty spondylitis is generally attended by manifestations in other parts of the body. He had failed to state that his patient had limited respiratory movements.

Dr. Vance related a case in which there was, in addition to the spinal affection, complete immobilization of the thorax with chiefly diaphragmatic respiration.

Dr. Bartow had seen a case in which relief was afforded by the spinal jacket.

Dr. Gillette reported a case which, at the first glance, resembled the deformity of Potts' disease, but which proved to be rachitic in its etiology. Improvement followed a few days after suspension was begun.

SACRO-ILIAC DISEASE.

Dr. Lee said the sequence of events is as follows: (1) Injury of the synchondrosis; (2) subacute inflammation; (3) irritation of the nerves of the joint, transmitted to the nearest plexus; (4) resulting pain in the sciatic. The sciatica should be considered the result, not the cause, of all the trouble. In nine cases out of ten, neuralgia is the effect and not the cause of any trouble. As stooping in sacro-iliac disease is injurious, he had devised a handy instrument with which the patient can pick up an object from the floor while remaining erect.

